# Sub-Saharan Africa's Mothers, Newborns, and Children: Where and Why Do They Die?

**DOI:** 10.1371/journal.pmed.1000294

**Published:** 2010-06-21

**Authors:** Mary V. Kinney, Kate J. Kerber, Robert E. Black, Barney Cohen, Francis Nkrumah, Hoosen Coovadia, Paul Michael Nampala, Joy E. Lawn, on behalf of the Science in Action: Saving the lives of Africa's mothers, newborns, and children working group

**Affiliations:** 1 Saving Newborn Lives/Save the Children, Cape Town, South Africa; 2 John Hopkins University Bloomberg School of Public Health, Baltimore, Maryland, United States of America; 3 United States National Academy of Sciences, Washington, DC, United States of America; 4 Ghana Academy of Arts and Science, Accra, Ghana; 5 The Universities of Kwazulu-Natal and the Witwatersrand, Durban, South Africa; 6 Uganda National Academy of Sciences, Uganda

## Abstract

In the first article in a series on maternal, newborn, and child health in sub-Saharan Africa, Joy Lawn and colleagues outline where and why deaths among mothers and children occur and what known interventions can be employed to prevent these deaths.

This paper is part of a *PLoS Medicine* series on maternal, newborn, and child health in Africa.

**Summary Points**

- Every year 4.4 million children—including 1.2 million newborns—and 265,000 mothers die in sub-Saharan Africa. This amounts to 13,000 deaths per day or almost nine deaths every minute. Sub-Saharan Africa has half of the world's maternal, newborn, and child deaths.
- The five biggest challenges for maternal, newborn, and child health in sub-Saharan Africa are: pregnancy and childbirth complications, newborn illness, childhood infections, malnutrition, and HIV/AIDS.
- Many scientifically proven health interventions are available for maternal, newborn, and child health such as medicines, immunizations, insecticide-treated bed nets, and equipment for emergency obstetric care. Yet many African governments are currently underutilizing existing scientific knowledge to save women's and children's lives.
- A scientific approach based on local epidemiological and coverage data is needed to prioritize the highest impact and most appropriate interventions in a given context.
- Most countries in sub-Saharan Africa are behind in achieving the Millennium Development Goals (MDGs) for maternal and child health by 2015. However, progress in several low-income countries demonstrates that the MDGs could still be attained through immediate strategic investments in selected evidence-based interventions and targeted health systems strengthening. Many countries are at a tipping point and now is the critical time to use local data to set priorities and accelerate action.

Nearly 4.7 million mothers, newborns, and children die each year in sub-Saharan Africa: 265,000 mothers die due to complications of pregnancy and childbirth [1]; 1,208,000 babies die before they reach one month of age [2]; and 3,192,000 children, who survived their first month of life, die before their fifth birthday [1]. This toll of more than 13,000 deaths per day accounts for half of the world's maternal and child deaths. In addition, an estimated 880,000 babies are stillborn in sub-Saharan Africa and remain invisible on the policy agenda [3].

With only five years left to achieve the United Nation's Millennium Development Goals (MDGs) for maternal and child health, most African countries in the region are currently unlikely to meet their MDG targets [4]. Since time is short for achieving success, a critical understanding of where and why these deaths occur, and of strategic, data-based prioritization of interventions, are essential to accelerate progress.

The aim of this paper is to present the current situation in sub-Saharan Africa for mothers, newborns, and children under age 5 years—including the progress towards the MDGs for maternal and child health, why and where deaths occur, what known interventions can be employed to prevent these deaths, and current coverage of these interventions. All data used in this review are from the most recent UN databases, national household surveys, and peer-reviewed papers where appropriate, which are referenced accordingly.

This paper is the first of two in a PLoS Medicine series on maternal, newborn, and child health (MNCH) in sub-Saharan Africa, along with three related essays providing critical commentary [5]–[8]. The papers are based on a report entitled “*Science in Action: Saving the Lives of Africa's Mothers, Newborns, and Children*,” which was developed for the annual meeting of the African Science Academy Development Initiative in Accra, Ghana, in November 2009 [9]. A team of over 60 scientists and researchers from nine countries outlined the current status of MNCH in sub-Saharan Africa, presented evidence-based solutions, and used national data to identify immediate high-impact opportunities for saving lives. With the MDG deadline of 2015 rapidly approaching, data on progress and on where, when, and why deaths occur are a critical basis for prioritizing actions.

## Progress towards Millennium Development Goals 4 and 5 in Sub-Saharan Africa

**Figure 1:**
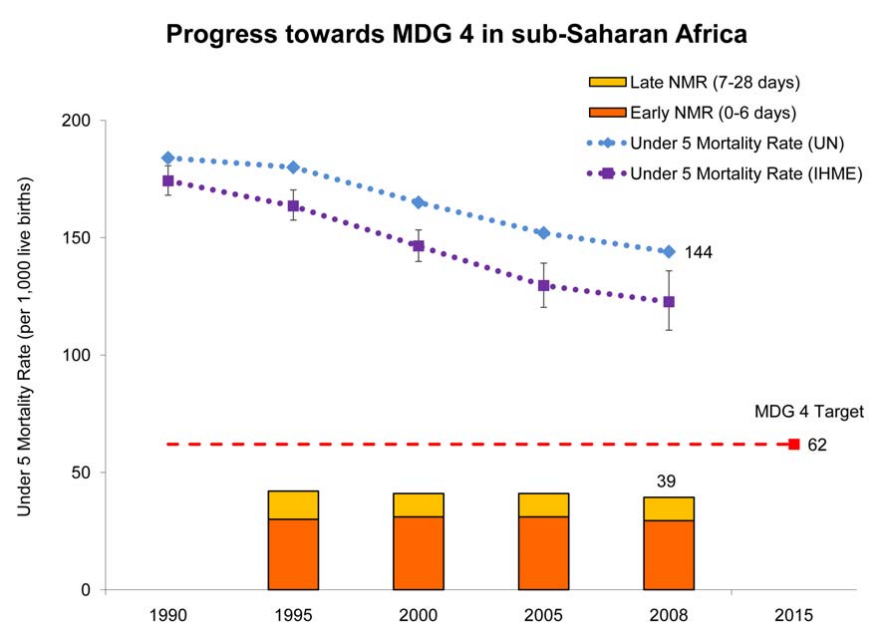
Progress towards Millennium Development Goal 4 for newborn and child survival in sub-Saharan Africa. U5MR has declined since 1990 in sub-Saharan Africa in relation to the MDG 4 target for child survival, a reduction of the U5MR by two-thirds by 2015. Although some reduction in the U5MR has been achieved, particularly since 2000, on average, the pace of the decline across the region has been too slow to meet the MDG 4 target. The figure also shows the regional trend for the NMR since 1995. Newborn deaths account for over a quarter of under-5 deaths and there has been little decline. Figure adapted from Kinney et al. 2009 [9] and Lawn and Kerber 2006 [11]. Data from http://www.childmortality.org and updated for 2008 using data from *Countdown to 2015 for Maternal, Newborn and Child Health* [2] and *State of the World's Children* 2010 [1]. The second line on the graph uses data generated by IHME from Rajaratnam et al. 2010 [10].

MDG 4 calls for a two-thirds reduction in the under-5 mortality rate (U5MR) between 1990 and 2015 (Figure 1). At a regional level, almost no advancement was made in reaching this goal during the 1990s; yet, since 2000 there has been some progress [4]. Estimates generated by the Institute for Health Metrics and Evaluation (IHME) also suggest increasing progress [10]. According to UN data, an average annual decline of 7% would now be needed to put sub-Saharan Africa on track for MDG 4 [4]. Newborn deaths, or babies that die in first 28 days of life, account for a quarter of child deaths, and the regional neonatal mortality rate (NMR) has not declined at the same rate as under-5 mortality over the last two decades [9]–[11]. Nevertheless, six countries are on track for MDG 4: Cape Verde, Eritrea, Mauritius, Seychelles, and, most recently, Botswana and Malawi [1]. Another substantial gain in child survival is a dramatic reduction in measles deaths as a result of improved immunization coverage [4]. These trends show promise, and sub-Saharan Africa may be at a tipping point for child survival.

**Figure 2:**
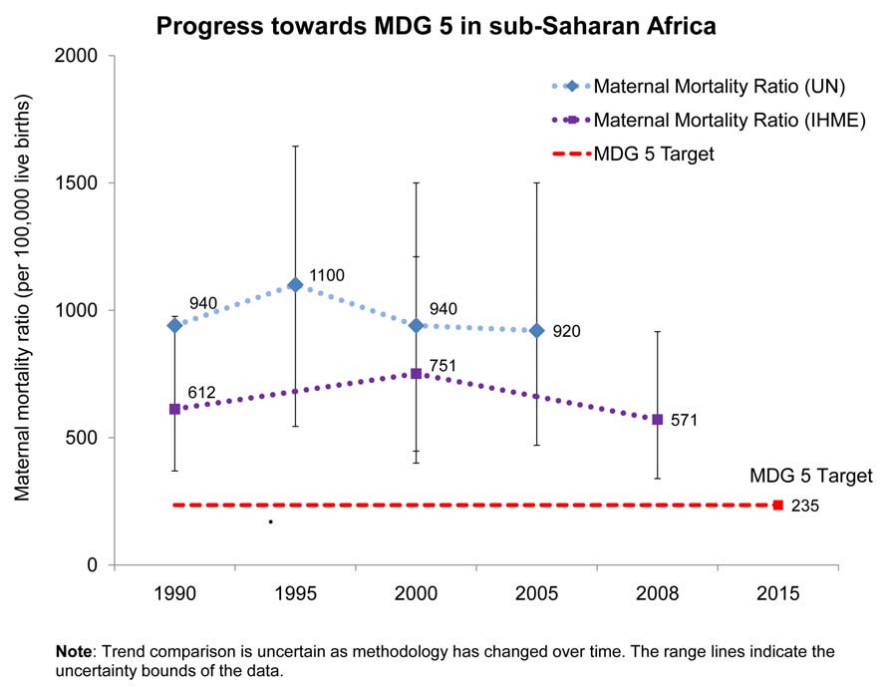
Progress towards Millennium Development Goal 5 for maternal survival in sub-Saharan Africa. MMR has remained practically unchanged since 1990 in sub-Saharan Africa in relation to the MDG 5 target for maternal survival, a reduction of the MMR by three quarters by 2015. However, the confidence intervals are extremely wide and trend comparison is uncertain as methodology has changed over time. Data for 1990 from WHO, UNICEF, UNFPA, and The World Bank 2007 [12]; data for 1995 are from WHO, UNICEF, and UNFPA 2001 [13]; data for 2000 are from WHO, UNICEF, UNFPA 2004 [14]; and data for 2005 are from Hill et al. 2007 [15]. The second line on the graph uses data generated by IHME from Hogan et al. 2010 [16].

With respect to MDG 5—to improve maternal health—the regional average maternal mortality ratio (MMR) has not changed with statistical significance since 1990 [12]–[16], as shown in Figure 2. However, because most modeled estimates have wide ranges of uncertainty and different methodologies, trend data should be interpreted with caution. When calculating the number of maternal deaths, the country-reviewed UN estimate for 2005 has been used [15]. Of the region's 46 countries, 40 are estimated to have high or very high maternal mortality (classified as MMR over 300 deaths per 100,000 live births) [15]. The proportion of deliveries attended by skilled health care personnel, one indicator of MDG 5 progress, has shown a minimal increase over the past few decades in sub-Saharan Africa, averaging 42% in 1990 and 46% in 2008 [4],[17].

Even though many of the regional average indicators are not encouraging, some individual countries are making progress. For example, Eritrea has achieved an average annual U5MR reduction of 5% since 1990 despite having one of the lowest gross national incomes per capita in the world [1]. Malawi, Tanzania, and Ghana are among countries with stagnant U5MR in the 1990s but have experienced up to a 30% decline in U5MR since 2000 [1]. Benin and Burkina Faso have registered increases in skilled birth attendance in the past ten years, by 18 and 12 percentage points respectively [18]. Ghana has also achieved an increase in skilled birth attendance associated with a policy of free medical care for pregnant women announced by the country's president in May 2008 and implemented through the National Health Insurance Scheme [19].

With several global and regional plans and commitments in place, there is renewed hope that maternal and child survival will continue to improve in the region. In recent years, global and national leaders have highlighted the importance of MNCH, such as the strategic framework for child survival and health-related MDGs presented by UN partners and adopted at an African Union meeting in 2005 [20], and the upcoming July 2010 Summit of the African Union on the theme “Maternal, Newborn and Child Health and Development in Africa.” Globally there is also more attention; for example the G8 leaders' statement “Promoting Global Health” in July 2009 [21], the global consensus statement for MNCH in September 2009 [22], and an announcement by Canada's Prime Minister that MNCH will be a top priority for the 2010 G8 meeting [23]. Importantly, between 2003 and 2006, donor investment for MNCH increased by 63% for child health and 66% for maternal and newborn health [24]. As a result, information on the current situation of maternal, newborn, and child health is critical to guide this action.

## Current Situation of Maternal, Newborn, and Child Health in Sub-Saharan Africa

**Figure 3:**
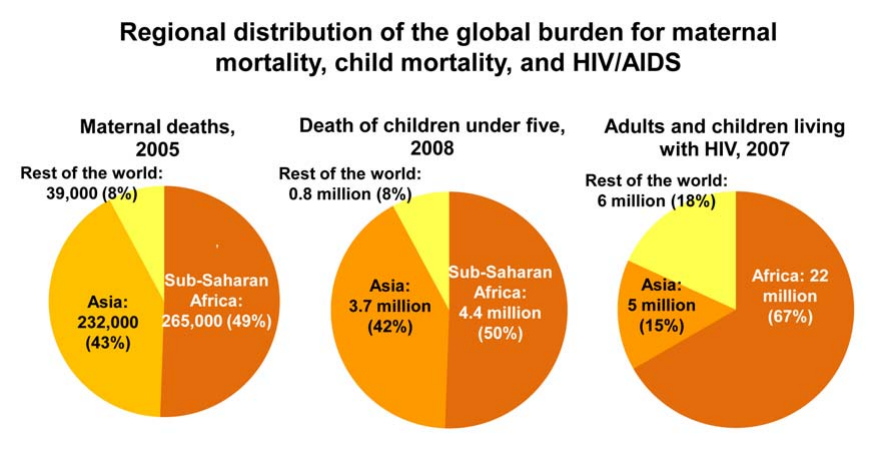
Regional distribution of the global burden for maternal mortality, child mortality, and HIV. Sub-Saharan Africa carries a high proportion of the global disease burden for maternal and child health, and HIV/AIDS. The region accounts for half of the world's maternal, newborn, and child deaths and two-thirds of people living with HIV/AIDS. Figure adapted from Kinney et al. 2009 [9]; data for maternal deaths in 2005 from Hill et al. 2007 [15]; data for under-5 child deaths in 2008 from *State of the World's Children 2010* [1]; data for number of adults and children living with HIV in 2007 from UNAIDS, *Report on the Global AIDS Epidemic, 2008* [26].

Sub-Saharan Africa accounts for 11% of the world's population yet half of the world's burden of maternal, newborn, and child deaths (Figure 3)—nearly 4.7 million deaths per year. The region is also carries a disproportionate share of other major health challenges: 90% of the world's malaria deaths [25], 67% of people living with HIV/AIDS globally [26], and 28% of the underweight children in developing countries [27]. Yet this heavy burden falls on the region with the lowest density of health care workers (see Box 1).

**Box 1. Many sub-Saharan African Countries Rely on Mid-Level Cadre Health Workers**

A shortage of qualified health workers is a major constraint for accessing essential health care in Africa, which suffers more than 24% of the global burden of disease, and yet has only 3% of the world's health workers [61]. Sub-Saharan Africa is the region with the lowest density of total health workers per 1,000 population of 2.3 compared to Europe with 18.9 [61]. At least 36 countries of the 46 countries experience critical shortages in human resources.

Due to this human resource shortage, many countries rely on task shifting. Task shifting presents a viable solution for improving health care coverage by making more efficient use of the human resources already available while longer training programs are expanded. However tasks should be selected, roles defined and supervision is critical.

Simpler tasks may be shifted to the lower level such as the use of extension workers or community health workers for example for immunization, contraceptive services or community case management of childhood illness. Some countries, such as Ghana, have used medical assistants to diagnose and treat common disorders for decades [62]. Since many developing countries have already successfully employed the use of mid-level health care workers, the current question is how to expand, supervise and monitor their role.

Alternatively more complex tasks can be delegated to mid level health worker cadres with appropriate training e.g. non-physician clinicians, midwives. For example, in Malawi, Mozambique, and Tanzania, around 90% of emergency obstetric operations, including caesarean sections are performed by clinical officers. Training more mid-level health workers especially in surgery will save lives at lower cost and with higher retention in hard to serve areas [63]. Yet in order to maximize the benefits that may accrue from building capacity of and using non-physicians, some concerns need to be addressed such as qualification levels, ethical conduct, and abuse of roles and low motivation. Recent studies suggest that some of these challenges can be resolved with salary enhancements and greater professional recognition [64]. In the long term, additional investment in training non-physician clinicians is needed generate a critical mass of skilled cadres who could stay at rural posts and serve at district hospitals long.

Within the region, countries in West and Central Africa generally have higher rates of maternal mortality and under-5 mortality than Eastern and Southern African countries. Nigeria alone, as the most populous country in the region and the eighth most populous country in the world, accounts for a quarter of all maternal, newborn, and child deaths in sub-Saharan Africa [1].

### Causes of Maternal, Newborn, and Child Deaths

There are five major challenges for maternal, newborn, and child health in sub-Saharan Africa: pregnancy and childbirth complications, newborn illness, childhood infections, malnutrition, and HIV/AIDS [9]. These need to be overcome for the region to achieve the MDGs for maternal and child survival. HIV/AIDS results in 210,000 child deaths each year in the region, but this burden falls mainly on 16 countries in Southern Africa, and in some of these HIV/AIDS is the major cause of death.

**Figure 4:**
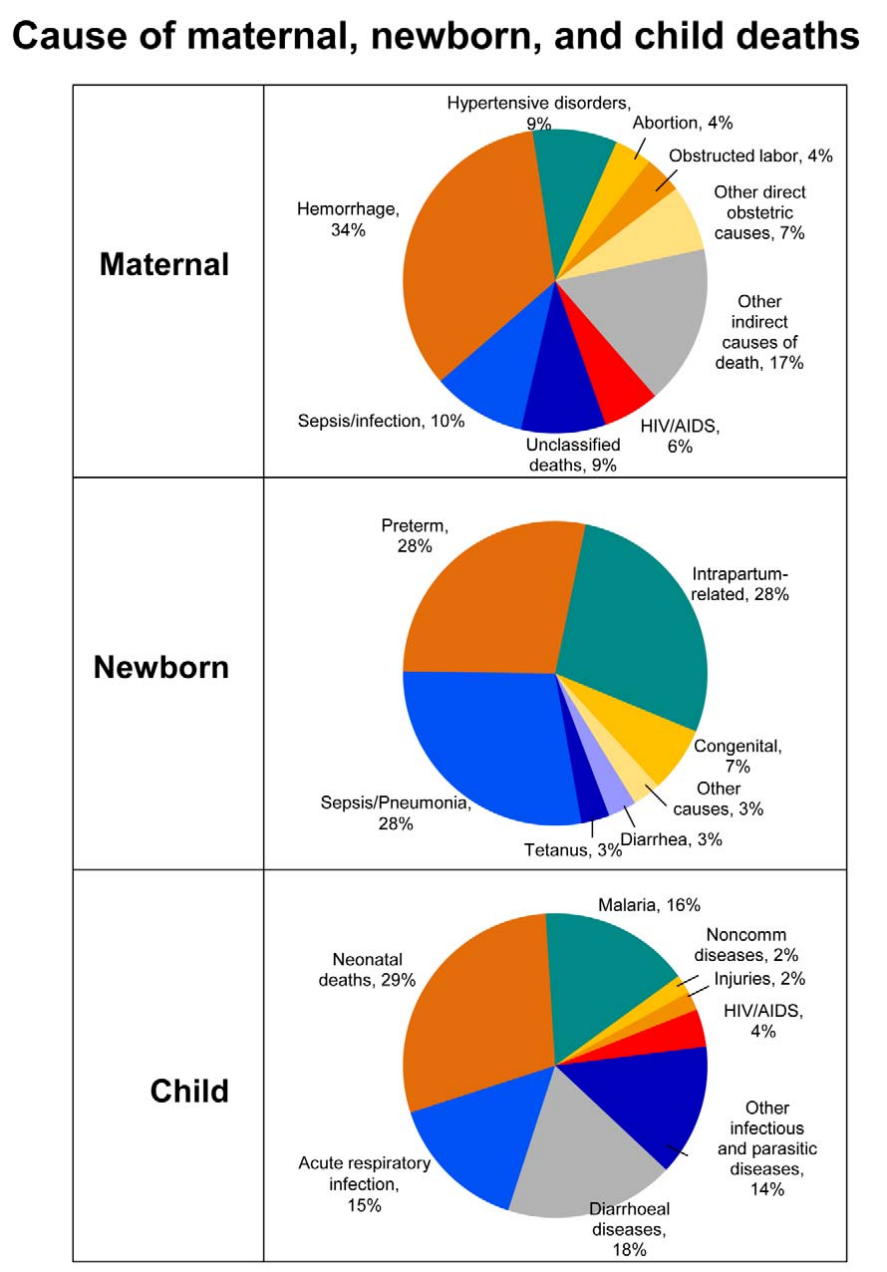
Causes of maternal, newborn, and child deaths in sub-Saharan Africa. More than half of maternal deaths in Africa are due to direct obstetric complications, with hemorrhage being the leading cause. Maternal sepsis and hypertensive disorders are important and preventable causes of maternal mortality. Newborn deaths account for more than one quarter of under-5 deaths in Africa. Infections are the biggest cause of newborn death yet the most feasible causes to prevent and treat. The two other major causes of newborn deaths are preterm birth complications and intrapartum-related (previously called “birth asphyxia”), which are closely linked to maternal health. Main causes of under-5 deaths include pneumonia, diarrhea, and malaria. Globally more than one-third of postneonatal child deaths are attributable to undernutrition. The cause-of-death profile varies between and within countries, with HIV/AIDS contributing to more deaths in southern African countries. Figure adapted from Kinney et al. 2009 [9] using data sources for maternal (Khan et al. 2006 [28]) and newborn and child (Black et al. 2010 [34]) causes of death.

According to the most recent World Health Organization (WHO) analysis, most maternal deaths in Africa are related to direct obstetric complications that occur around the time of childbirth—mainly hemorrhage, hypertension, sepsis, and obstructed labor, which combined account for 64% of all maternal deaths (Figure 4) [28]. Non-pregnancy related infections, such as HIV/AIDS and pneumonia, account for 23% of the deaths and unsafe abortion accounts for 4% of maternal deaths in Africa [28]. More than half of maternal deaths take place within one day of birth [29]. Malnutrition, including maternal anemia, iodine deficiency, and poor-quality diet, also contribute to maternal mortality and the high incidence of stillbirths and congenital abnormalities [27]. HIV-infected mothers' risk of dying is ten times higher than that of HIV-negative mothers [30].

It is estimated that 880,000 stillbirths occur each year in sub-Saharan Africa [3], yet there is limited attention to these deaths [31]. Nearly one-third of all stillbirths occur during labor and are difficult to distinguish from early neonatal deaths [3],[32]. Many of these deaths are preventable with the same solutions that would save many mothers and newborns [33].

With mortality in later childhood decreasing, the proportion of deaths that take place in the neonatal period has been rising, with three main causes accounting for 88% of newborn deaths in the region: (1) infections (including sepsis/pneumonia, tetanus, and diarrhea); (2) intrapartum-related conditions (“birth asphyxia”); and (3) preterm births [34]. Up to 90% of newborns who die are low birthweight (<2,500 grams) including preterm babies, who have the greatest risk of death. Yet most could be saved with simple care such as warmth, feeding, hygiene, and early treatment of infections [35].

After the first month of life, two-thirds of child deaths are due to pneumonia, diarrhea, and malaria, which are preventable and also very feasible to treat [34]. Undernutrition contributes to child mortality by increasing children's risk of dying from infections. With over 31 million African children underweight, the nutritional risk factors—including vitamin A and zinc deficiencies and suboptimal breastfeeding—contribute to more than one-third of postneonatal child deaths [27].

While the five major causes of maternal, newborn, and child deaths are similar across countries, the proportions vary, especially for those countries more affected by HIV/AIDS. For example, even though HIV/AIDS accounts for only 4% of child deaths in the region overall, in South Africa, more than half of child deaths are due to HIV/AIDS and at least 38% of maternal deaths are from HIV/AIDS, tuberculosis, and pneumonia [30].

### Other Factors That Influence Maternal, Newborn, and Child Heath

In addition to the direct causes of deaths, poverty and inequity undermine the survival of mothers, newborns, and children. Intersectoral actions such as expanding educational opportunities, improving living and working conditions, and increasing access to water and sanitation could dramatically improve health outcomes within even one generation. [36].

Poverty is an underlying cause for many deaths, with nearly 99% of global maternal and newborn deaths occurring in low- and middle-income countries [37]. Maternal mortality is more than twice as high in the poorest households than among the least poor households [29]. Poverty undermines MNCH through numerous pathways, including increased risk of illness and undernutrition through insufficient diet, inadequate housing and sanitation, and reduced care-seeking and access to health care services.

Gender discrimination, low levels of female education, and lack of empowerment prevent women from seeking care, having the autonomy to make decisions, and accessing the best choices for themselves and their children's health, resulting in critical delays and unnecessary deaths. Educated women are less likely to die in childbirth, and children whose mothers have a primary school education are half as likely to die before age five as children whose mothers have no education [11]. Specific health education for families and mothers-to-be are key components to MNCH. Shifting harmful norms that disempower women, for example, ending female genital mutilation, are also critical for improving MNCH outcomes [38].

The urban/rural divide also affects MNCH and access to health care. Mortality is consistently lower in urban areas than in rural areas, with remote communities often having poorer access to health care [39]. However, rapid urbanization is associated with crowded living conditions, poor sanitation, and widespread poverty. Thus, even these urban averages mask disparities for the fast-growing population of urban and peri-urban poor across the continent who struggle as much as or more than their rural counterparts to access quality health care.

Countries experiencing conflict also tend to have higher rates of maternal, newborn, and child death due to unstable institutions and weak health systems. Most of the ten countries in sub-Saharan Africa with the highest mortality rates have seen recent complex emergencies including the Democratic Republic of the Congo, Angola, Liberia, Sierra Leone, and others. One study in the Democratic Republic of the Congo found that maternal deaths were more common in the conflict-riddled eastern provinces, 1,174 maternal deaths per 100,000 live births, compared to in the west where the rate was 811 deaths per 100,000 live births [40].

Complex emergencies, such as conflict and natural disasters, present considerable challenges to delivering MNCH services and maintaining a functional health system. These situations are often marked by a lack of equipment and supplies, poor referral systems, bad and worsening conditions of health facilities, loss of human resources for health, and deteriorating transportation networks. Corruption, authoritarian regimes, weak institutions, and limited freedoms can also inhibit access to effective care for mothers, newborns, and children. Conversely, good governance is linked to systematic progress towards comprehensive and effective health systems [36].

Finally, health care is simply unaffordable for many families in sub-Saharan Africa. User fees and cost-sharing arrangements remain a major barrier to accessing health services, especially for the poor. Other economic barriers include informal health care fees, the cost of medicines and tests not supplied in public health facilities, the cost of not working during hospitalization, travel, food, and accommodations. Although removing fees might benefit poor families and increase health service utilization, it requires careful planning, management, and support by other policy measures to ensure that quality of care is maintained and health facility funding needs are met through sources other than user fees [41]. Ghana, South Africa, and Uganda have all experienced some success in user fee elimination for MNCH services [18],[42],[43].

## Solutions for Maternal, Newborn, and Child Health: Interventions and Health Packages

A number of reviews published over the past seven years have looked at interventions to reduce child [44],[45], newborn [46], perinatal, and maternal mortality [47]; to address intrapartum-related deaths and stillbirths [31],[33],[48]; to improve sexual and reproductive health [49] and child development [50]; and to reduce maternal and childhood undernutrition [51] (see Table S1) [52].

While single or vertical interventions can be critical in rapidly increasing coverage, a more sustainable solution is to integrate effective interventions and delivery strategies within existing health system packages [53]. Bridging the artificial divide between vertical approaches (that focus on specific donor agendas, disease priorities, and interventions) and horizontal ones (that aim to strengthen the overall structure and functions of the health system), may increase the efficiency of service delivery and build a results-focused health system [54]. There is increasing evidence to suggest that when MNCH interventions are packaged and provided through various service delivery modes tailored to suit existing health systems, cost-effectiveness is enhanced and available human resources are maximized [53].

**Figure 5:**
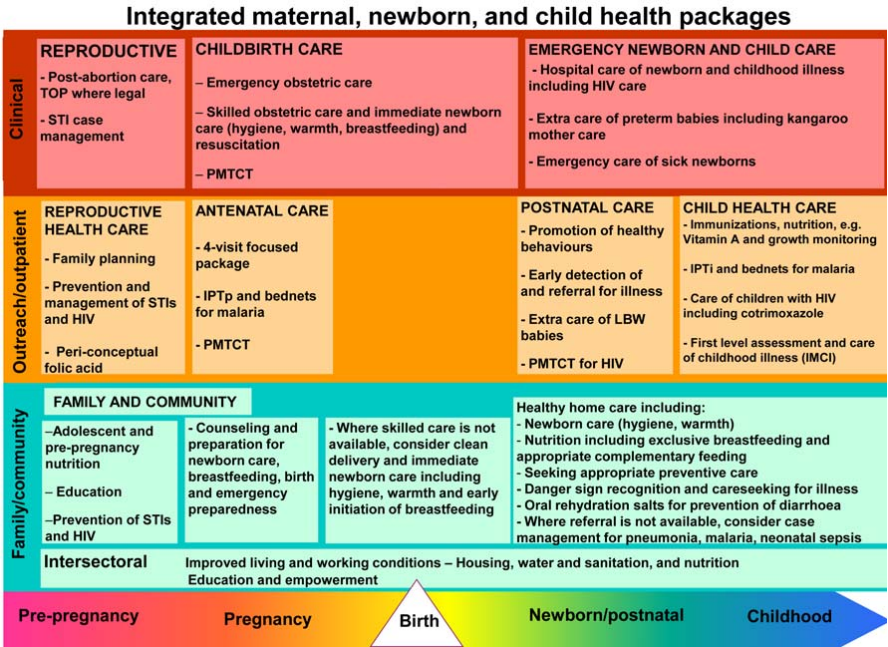
Integrated maternal, newborn and child health packages. Eight integrated packages for MNCH, with evidence-based interventions along the continuum of care, organized by lifecycle and place of service delivery. Figure from Kinney et al. 2009 [9] adapted from Kerber et al. 2007 with permission [53].

The continuum of care is a core organizing principle for health systems that emphasizes linkages between health care packages across time and through various service delivery strategies. An effective continuum of care addresses the needs of the mother, newborn, and child throughout the life cycle wherever care is provided: at home, the primary care level, and at district and regional hospitals [53]. Eight basic health packages are present in almost every health system that make up the continuum of care (Figure 5):

1. Clinical care package for reproductive health;
2. Clinical care package for childbirth;
3. Clinical care package for newborn and child;
4. Outpatient and outreach package for reproductive health care;
5. Outpatient and outreach package for antenatal care;
6. Outpatient and outreach package for postnatal care;
7. Outpatient and outreach package for child health care; and
8. Family and community care package.

A functioning continuum of care for MNCH relies on these integrated health packages to deliver a range of high-impact interventions (see Figure 5). Although these packages exist in nearly all settings, low- and middle-income countries cannot possibly scale up and implement all MNCH interventions within these packages at once, so priorities have to be selected. Packages can be designed based on simpler, specific interventions to achieve a particular outcome, and then become more complex in number and types of interventions over time according to local needs and capacity. The rate of scale-up depends on the functionality of the health system—human resource capacity, health-facility infrastructure, supply systems, financial resources, government stewardship, district-level management, and monitoring.

## Current Coverage, Equity, and Quality Gaps

**Figure 6:**
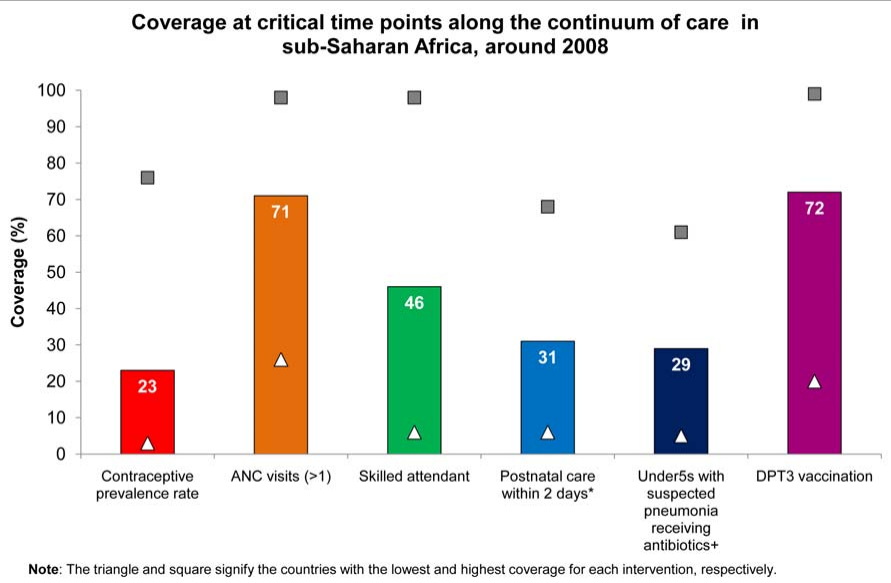
Coverage at critical time points along the continuum of care in sub-Saharan Africa, around the year 2008. The regional average coverage varies along the continuum of care for MNCH in sub-Saharan Africa. Currently, there are data available for six of the eight basic service delivery packages. The reproductive health packages delivered through outpatient/outreach services is represented by contraceptive prevalence rate. ANC package delivered through outpatient/outreach service is represented by one ANC visit. Childbirth clinical care package is represented by skilled attendant at birth. Postnatal care package delivered through outpatient/outreach service is represented by a postnatal check on the mother's health within 2 days of childbirth. Newborn baby and child clinical care package is represented by under-5 children with suspected pneumonia receiving antibiotics. Child health package delivered through outpatient or outreach service is represented by three doses of DPT vaccine. The reproductive health clinical care package and family and community care package have no routine indicator data available. *Postnatal care data from 12 countries. ^+^Under-5 children with suspected pneumonia receiving antibiotics data from 20 countries. Figure adapted from Kinney et al. 2009 [9] with data from a new analysis of Demographic and Health Surveys (2005–2008) and *State of the World's Children 2010* [1].

Coverage for the basic service delivery packages for MNCH in sub-Saharan Africa varies across the continuum of care. The eight basic packages consist of multiple interventions (Figure 5), and Figure 6 shows the regional coverage data for one representative indicator or contact point for six of the eight packages. There are currently no routine indicator data available for the reproductive health clinical care package or for the family and community care package.

The *coverage gap* is the difference between current coverage and full or universal coverage that reaches all families with essential care. Services that can be scheduled—notably antenatal care (ANC) and immunization—tend to have relatively high coverage across the region with 71% of pregnant women receiving at least one ANC visit with a skilled attendant and 72% of children receiving the required three doses of the vaccine against diphtheria, pertussis, and tetanus (DPT) [1]. However, cases that require 24-hour curative services—such as skilled attendance and emergency obstetric care, and case management for pneumonia, diarrhea, and malaria—have much lower coverage [2]. Less than 50% of births are attended by skilled personnel, and coverage of routine postnatal care for mothers and babies is very low (31%), partly because this is a recently recognized package with varying delivery strategies [55]. For children under 5 years of age, coverage of antibiotics for pneumonia is 29% [1]. Critical interventions such as contraception and postnatal care are possible through outreach but have not been given consistent policy priority. Figure 6 also shows the wide range of coverage for these packages among countries with the lowest and highest coverage levels marked. For example, skilled attendance at birth varies from 6% in Ethiopia to 98% in Mauritius, and postnatal care is ten times higher in Ghana than in Chad [2]. Generally, overall progress for scale-up of high-impact MNCH interventions has been slow in sub-Saharan Africa, with some notable exceptions such as insecticide-treated nets and immunizations, which have received more attention [2].

The *equity gap*—the difference between the care received by the richest families compared to the poorest families—is hidden by national averages. Equitable care involves providing care to all families according to need, rather than according to income or other social grouping. Large disparities exist between rich and poor people and areas, public and private health sectors, provinces or districts, and among rural, urban, and periurban populations. Even for some primary health interventions with high coverage, such as immunizations, coverage is lower for poorer families. For clinical and curative care, the gap between access to care for the richest and poorest households is much wider. For example, skilled attendance during childbirth is 5-fold higher for the richest families than the poorest [33]. Increased investment to improve equitable access to care and targeting the poorest and hardest-to-reach areas must be systematically improved to reach all families, particularly during childbirth and the critical early postnatal period.

**Figure 7:**
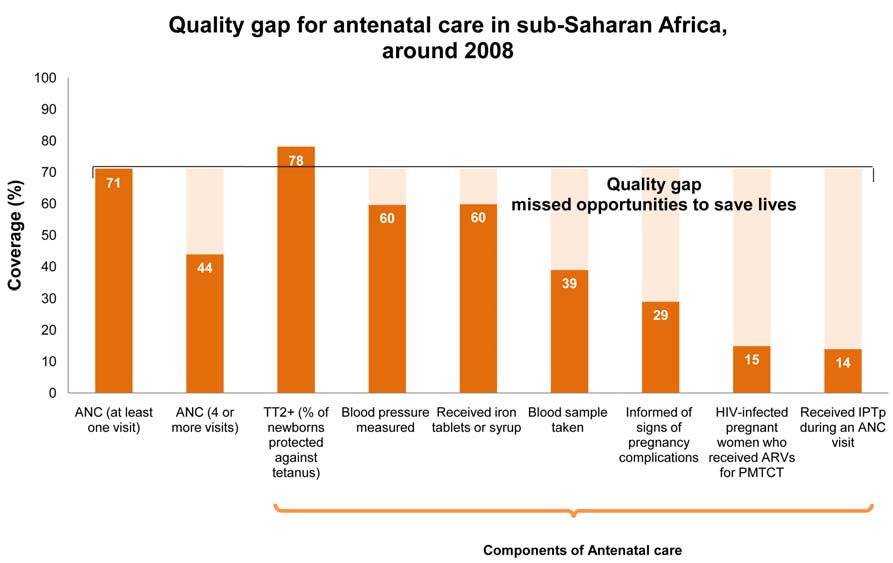
Quality gap for antenatal care in sub-Saharan Africa, around the year 2008. There is a substantial quality gap in ANC services in sub-Saharan Africa. While coverage of at least one ANC visit is relatively high at 71% compared to other MNCH services (see Figure 6), many women attending ANC do not receive the full range of evidence-based components during pregnancy. This quality gap demonstrates key missed opportunities within health systems. Tetanus vaccine coverage is higher because of outreach campaigns. Figure adapted from Kinney et al. 2009 [9] with a new analysis of data from Demographic and Health Surveys (2005–2008) and the UNAIDS *Report on the Global AIDS Epidemic, 2008* [26], and *State of the World's Children 2010* [1].

The *quality gap* is the difference between coverage of the basic package and provision of effective and client friendly care. To save the most lives, increasing coverage of care alone is not enough. Quality must improve and remain high in order to provide effective care and to maintain demand for health services. Quality service provision requires the availability of people with appropriate skills and the essential equipment and drugs. For example, the contact point of one ANC visit is not as effective as the full package of at least four ANC visits with evidence-based content including the identification of high-risk pregnancies, counseling for birth preparedness, and testing and treating for illnesses such as HIV/AIDS and syphilis. Figure 7 shows the regional coverage of at least one antenatal visit (71%), with far fewer women who attend ANC receive the full range of evidence-based interventions during pregnancy, thus, missing key opportunities to provide quality care. Gaps in measurement of quality of care also affect the ability to identify and reduce such quality gaps. Quality care at birth, especially provision of cesarean section and neonatal resuscitation, are sensitive indicators of health system quality and performance [56].

As newborn health has come to global attention only recently, some key high-impact innovations are still not included in routine programs. One example is Kangaroo Mother Care—a simple technique in which the baby is tied to the mother's front, providing warmth, increased feeding, reduced infections, and more rapid recognition of illness. New evidence shows that hospital-based Kangaroo Mother Care reduces deaths for babies under 2,000 grams by 51% [57]. An important area of research is around whether Kangaroo Mother Care can be safely initiated at the community level for families lacking access to health facilities.

For African countries with a high burden of HIV/AIDS, there continue to be many opportunities for prevention of mother-to-child transmission and improving the coverage, quality and equity of available services. New data suggest that breastfeeding (which saves many lives, including reducing non-HIV deaths) can now be made safe for HIV-positive mothers and their babies. Exclusive breastfeeding for six months with antiretroviral drugs minimizes transmission of HIV infection [58]–[60].

Our analysis of the progress for MDGs 4 and 5 as well as coverage, equity, and quality gaps can inform governments and health policy makers of the current status and where care is lacking, but planning for the most effective course of action and where investment would save the most lives requires further analysis of lives saved and cost. [5].

## Conclusion: Identifying and Investing in Priority MNCH Interventions

With nearly 4.7 million mothers, newborns, and children dying each year in sub-Saharan Africa, and only five years left for achieving the MDGs for maternal and child health, the need for immediate action is clear. If essential evidence-based MNCH interventions reached all families in the region by 2015, nearly four million lives could be saved each year [5]. The potential is great and the evidence, together with unprecedented new investment in maternal and child health from continental leaders and increasingly from development partners [21], offers new hope for the future.

Progress in several low-income countries demonstrates that the MDGs for maternal and child survival could still be attained through immediate strategic investments and targeted health systems strengthening, but this effort requires the use of the best national and sub-national mortality and health service coverage data to prioritize interventions that would be most likely to reduce mortality, including the use of lives-saved analysis and costing as discussed in another paper in this series [5]. Many countries in sub-Saharan Africa are at a tipping point for achieving the MDGs for maternal and child survival, but will applied science lead to evidence-based policy decisions and implementation, or will this critical momentum be wasted? We challenge leaders both inside and outside Africa, and especially from the African Union Summit in 2010, to ensure that science moves to action for Africa's mothers, newborns, and children.

## Supporting Information

Table S1 Recent reviews of interventions that potentially impact maternal, newborn, and child health and nutrition. Previously published series and supplements that have assessed and analyzed interventions and strategies relating to MNCH. Adapted and updated from Bhutta et al. 2008 [52].
(0.46 MB TIF)

## Abbreviations

ANC: antenatal care
IHME: Institute for Health Metrics and Evaluation
MDG: Millennium Development Goal
MMR: maternal mortality rate
MNCH: maternal, newborn and child heath
NMR: neonatal mortality rate
U5MR: under 5 mortality rate
WHO: World Health Organization
