# Supplementary figures and images for: Sub-Saharan Africa's Mothers, Newborns, and Children: Where and Why Do They Die?
Source: PLoS Med. 2010 Jun 21;7(6):e1000294. doi: 10.1371/journal.pmed.1000294 (PMC2888581; doi:10.1371/journal.pmed.1000294)

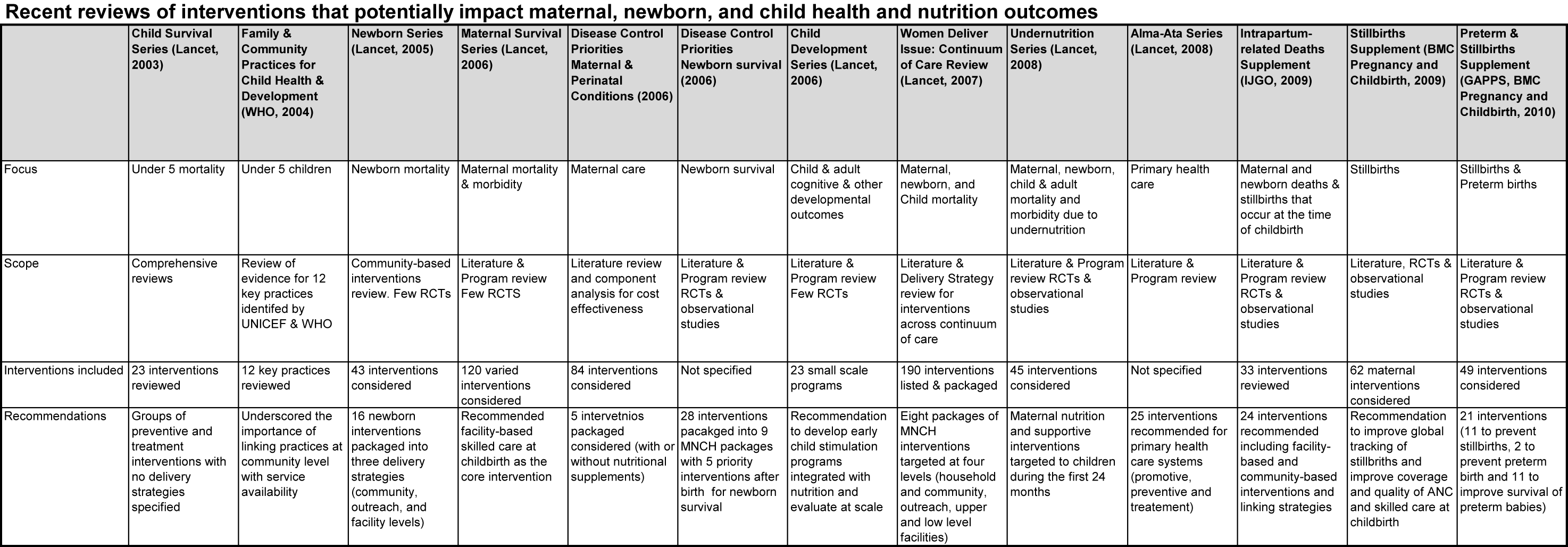

Supplement: Table S1 — Recent reviews of interventions that potentially impact maternal, newborn, and child health and nutrition. Previously published series and supplements that have assessed and analyzed interventions and strategies relating to MNCH. Adapted and updated from Bhutta et al. 2008 [52]. (0.46 MB TIF) [file pmed.1000294.s001.tif]
